# Whole Genome Sequence of the *Treponema pallidum* subsp. *endemicum* Strain Bosnia A: The Genome Is Related to Yaws Treponemes but Contains Few Loci Similar to Syphilis Treponemes

**DOI:** 10.1371/journal.pntd.0003261

**Published:** 2014-11-06

**Authors:** Barbora Štaudová, Michal Strouhal, Marie Zobaníková, Darina Čejková, Lucinda L. Fulton, Lei Chen, Lorenzo Giacani, Arturo Centurion-Lara, Sylvia M. Bruisten, Erica Sodergren, George M. Weinstock, David Šmajs

**Affiliations:** 1 Department of Biology, Faculty of Medicine, Masaryk University, Brno, Czech Republic; 2 The Genome Institute, Department of Genetics, Washington University School of Medicine, St. Louis, Missouri, United States of America; 3 Department of Medicine, University of Washington, Seattle, Washington, United States of America; 4 Public Health Service GGD Amsterdam, Amsterdam, The Netherlands; Beijing Institute of Microbiology and Epidemiology, China

## Abstract

**Background:**

*T. pallidum* subsp. *endemicum* (TEN) is the causative agent of bejel (also known as endemic syphilis). Clinical symptoms of syphilis and bejel are overlapping and the epidemiological context is important for correct diagnosis of both diseases. In contrast to syphilis, caused by *T. pallidum* subsp. *pallidum* (TPA), TEN infections are usually spread by direct contact or contaminated utensils rather than by sexual contact. Bejel is most often seen in western Africa and in the Middle East. The strain Bosnia A was isolated in 1950 in Bosnia, southern Europe.

**Methodology/Principal Findings:**

The complete genome of the Bosnia A strain was amplified and sequenced using the pooled segment genome sequencing (PSGS) method and a combination of three next-generation sequencing techniques (SOLiD, Roche 454, and Illumina). Using this approach, a total combined average genome coverage of 513× was achieved. The size of the Bosnia A genome was found to be 1,137,653 bp, i.e. 1.6–2.8 kbp shorter than any previously published genomes of uncultivable pathogenic treponemes. Conserved gene synteny was found in the Bosnia A genome compared to other sequenced syphilis and yaws treponemes. The TEN Bosnia A genome was distinct but very similar to the genome of yaws-causing *T. pallidum* subsp. *pertenue* (TPE) strains. Interestingly, the TEN Bosnia A genome was found to contain several sequences, which so far, have been uniquely identified only in syphilis treponemes.

**Conclusions/Significance:**

The genome of TEN Bosnia A contains several sequences thought to be unique to TPA strains; these sequences very likely represent remnants of recombination events during the evolution of TEN treponemes. This finding emphasizes a possible role of repeated horizontal gene transfer between treponemal subspecies in shaping the Bosnia A genome.

## Author Summary

Uncultivable treponemes represent bacterial species and subspecies that are obligate pathogens of humans and animals causing diseases with distinct clinical manifestations. *Treponema pallidum* subsp. *pallidum* causes sexually transmitted syphilis, a multistage disease characterized in humans by localized, disseminated, and chronic forms of infection, whereas *Treponema pallidum* subsp. *pertenue* (agent of yaws) and *Treponema pallidum* subsp. *endemicum* (agent of bejel) cause milder, non-venereally transmitted diseases affecting skin, bones and joints. The genetic basis of the pathogenesis and evolution of these microorganisms are still unknown. In this study, a high quality whole genome sequence of the *T. pallidum* subsp. *endemicum* Bosnia A strain was obtained using a combination of next-generation sequencing approaches and compared to the genomes of available uncultivable pathogenic treponemes. Relative to all known genomes of *Treponema pallidum* subspecies, no major genome rearrangements were found in the Bosnia A. The Bosnia A strain clustered with other yaws-causing strains, while syphilis-causing strains clustered separately. In general, the Bosnia A genome showed similar genetic characteristics to yaws treponemes but also contained several sequences thought to be unique to syphilis-causing strains. This finding suggests a possible role of repeated horizontal gene transfer between treponemal subspecies in shaping the Bosnia A genome.

## Introduction

Uncultivable human pathogenic treponemes include *T. pallidum* subsp. *pallidum* (TPA), causing syphilis, *T. pallidum* subsp. *pertenue* (TPE), causing yaws, and *T. pallidum* subsp. *endemicum* (TEN), causing bejel, which is also known as endemic or nonvenereal syphilis. Infections caused by TPE and TEN are commonly denoted as endemic treponematoses. While yaws is found in warm, moist climates, bejel is found in drier climates. In both cases, infection is spread by direct contact (e.g. skin-to-skin or skin-to-mucosa). In addition, bejel can also be transmitted by contact with contaminated utensils [1], [2]. The current, and widespread, belief that yaws and bejel are non-sexually transmitted may simply reflect that these diseases mostly affect children that have not reached sexual maturity [3], [4].

Diagnosis of endemic treponematoses comprises clinical symptoms, epidemiological data, and serology. Since there is significant clinical similarity between the symptoms of syphilis and endemic treponematoses, and serology cannot discriminate between infection with TPA, TPE, and TEN strains, the epidemiology plays a major role in establishing a diagnosis. While yaws remains endemic in poor communities in Africa, Southeast Asia, and the western Pacific, bejel is predominant in western Africa and in the Middle East (reviewed in [2], [4]). Imported cases of yaws and bejel have been documented in children in Europe and Canada [5], [6]. With the accumulation of genetic data, molecular targets that can be used to differentiate treponemal subspecies, at the molecular level, have become available [2].

Endemic syphilis has been described almost everywhere in Europe since the 16th century (for review see [7]) and often was described under different names, e.g. the disease that appeared in Brno, CZ in 1575 was called *morbus Brunogallicus*, although it is not clear whether this infection was not perhaps caused by the syphilis treponeme [8]. The Bosnia A strain was isolated in 1950 in Bosnia, a country in southern Europe, from a 35-year old male with mucous patches under the tongue and on the tonsils; additionally, the patient showed secondary lesions (papules) on the face, trunk and extremities. Material for experimental inoculation of laboratory animals was taken from an ulcer on the shaft of the penis [9]. Although several other isolates were collected from bejel patients, only one additional strain of *T. pallidum* subsp. *endemicum* (Iraq B) is currently propagated in laboratory settings.

In this study, the complete genome sequence of the *T. pallidum* subsp. *endemicum* Bosnia A strain was obtained using a combination of next-generation sequencing approaches and compared to the genomes of the four TPE strains (Samoa D, CDC-2, Gauthier, Fribourg-Blanc isolate) and five TPA strains (Nichols, DAL-1, Chicago, SS14, Mexico A), all of which have been determined in recent years [10]–[15].

## Materials and Methods

### Amplification of TEN Bosnia A DNA

Bosnia A DNA was provided by Dr. Sylvia M. Bruisten from the Public Health Service, GGD Amsterdam, Amsterdam, The Netherlands. Bosnia A genomic DNA was amplified using the pooled segment genome sequencing (PSGS) method as described previously [11], [15]. Briefly, Bosnia A DNA was amplified with 214 pairs of specific primers to obtain overlapping PCR products (Table S1). To facilitate sequencing of paralogous genes containing repetitive sequences, PCR products were mixed in equimolar amounts into four distinct pools. Prior to next-generation sequencing (454-pyrosequencing, Illumina and SOLiD), the PCR products constituting each pool were labeled with multiplex identifier (MID) adapters and sequenced as four different samples. Two genomic regions were not amplified during PSGS and therefore were not used for sequencing the whole genome (gaps between coordinates 332290–335395 and 1123251–1123648 according to the Nichols sequence, AE000520.1 [16]; see Table S1). Sequences in these regions were Sanger sequenced at the University of Washington in Seattle (WA), USA.

### DNA sequencing and assembly of the Bosnia A genome

Whole genome DNA sequencing was done using the Applied Biosystems/SOLiD 3 System platform (Life Technologies Corporation, Carlsbad, CA, USA) combined with the Roche/Genome Sequencer FLX Titanium platform (454 Life Sciences, Branford, CT, USA) and with the Illumina/Solexa HiSeq 2000 approach (Illumina, San Diego, CA, USA). SOLiD sequencing was performed at SeqOmics Ltd (Mórahalom, Hungary), 454-pyrosequencing and Illumina sequencing were performed at The Genome Institute, Washington University School of Medicine (St. Louis, MO, USA). SOLiD, 454, and Illumina sequencing resulted in average read lengths of 40 bp, 504 bp and 100 bp and the total average depth coverage of 234×, 138× and 141×, respectively. 454 and Illumina sequencing reads were obtained from 4 distinct pools (sequenced as 4 different samples – see Table S1) and were separately assembled *de novo* using a Newbler assembler (454 Life Sciences, Branford, CT, USA) or TIGRA [17], respectively. The resulting 454 and Illumina contigs obtained for each pool were then aligned to the corresponding sequences (representing each pool sequence) of the reference CDC-2 genome (CP002375.1 [11]) using Lasergene software (DNASTAR, Madison, WI, USA). All gaps and discrepancies between these platforms within each pool were resolved using Sanger sequencing. Altogether, 20 genomic regions of the Bosnia A genome were amplified and Sanger sequenced. The final overlapping pool sequences were joined to obtain complete genome sequence of the Bosnia A strain. The SOLiD sequencing results were mapped to the reference Samoa D genome (CP002374.1 [11]) using the CLC Genomics Workbench (CLC bio, Cambridge, MA, USA) and were processed as mentioned above. The genome sequence obtained from SOLiD was then compared with the consensus genome sequence obtained from 454 and Illumina. All discrepancies were resolved using Sanger sequencing. Two TPE genomes (CDC-2 or Samoa D) were used as reference genomes for contig alignments since only few minor genetic differences have been found to be specific within individual TPE strains [11].

Due to low coverage, one genomic region (*Treponema pallidum* interval; TPI), was amplified with specific primers using a GeneAmp XL PCR Kit (Applied Biosystems, Foster City, CA, USA) [18], [19]. This TPI-48 interval contained paralogous genes *tprI* and *tprJ*. The PCR product was purified using a QIAquick PCR Purification Kit (QIAGEN, Valencia, CA, USA) according to the manufacturer's instructions and Sanger sequenced using internal primers. The *tprK* (TENDBA_0897), *arp* (TENDBA_0433), and TENDBA_0470 genes were amplified and cloned into the pCR 2.1-TOPO cloning vector (Invitrogen, Carlsbad, CA, USA). Nine independent clones for the *tprK* and *arp* genes and seven clones for TENDBA_0470 were sequenced as previously described [11]. A total of 7 genomic regions (in genes TENDBA_0040, TENDBA_0348, TENDBA_0461, TENDBA_0697, TENDBA_0859, TENDBA_0865 and TENDBA_0966) revealed intra-strain variability in the length of homopolymeric (G- or C-) stretches. The prevailing length of these regions was determined by TOPO TA-cloning and Sanger sequencing. At least five independent clones were sequenced as previously described [15].

### Gene identification, annotation and classification

The final whole genome sequence of the Bosnia A strain was assembled from SOLiD, 454 and Illumina contigs. In addition, Sanger sequencing was used for finishing the complete genome sequence and for additional sequencing including paralogous, repetitive and intra-strain variable chromosomal regions. Geneious software v5.6.5 [20] was used for gene annotation based on the annotation of the TPE CDC-2 genome [11]. Genes were tagged with TENDBA_ prefix. The original locus tag numbering corresponds to the tag numbering of orthologous genes annotated in the TPE CDC-2 genome [11]. The TENDBA_0897 gene, coding for TprK, showed intra-strain variable nucleotides and therefore nucleotides in variable regions were denoted with Ns in the complete Bosnia A genome. For proteins with unpredicted functions, a gene size limit of 150 bp was applied. Protein domains and functional annotation of analyzed genes were characterized using Pfam [21], CDD [22] and KEGG [23] databases.

### Comparisons of whole genome sequences

Whole genome nucleotide alignments of five TPA strains, four TPE strains and the Bosnia A strain were used for determination of genetic relatedness using several approaches including calculation of nucleotide diversity (π) and construction of a phylogenetic tree. All positions containing indels in at least one genome sequence were omitted from the analysis. There were a total of 1,128,391 nucleotide positions aligned in the final dataset. TPA strains comprised Nichols (re-sequenced genome CP004010.2 [14]), DAL-1 (CP03115.1 [13]), SS14 (re-sequenced genome CP004011.1 [14]), Chicago (CP001752.1 [10]), and Mexico A (CP003064.1 [12]) genomes, while TPE strains included Samoa D (CP002374.1 [11]), CDC-2 (CP002375.1 [11]), Gauthier (CP002376.1 [11]) and Fribourg-Blanc (CP003902.1 [15]). Whole genome alignments were constructed using Geneious software [20] and SeqMan software (DNASTAR, Madison, WI, USA). Nucleotide differences among studied whole genome alignments were analyzed using *DnaSP* software, version 5.10 [24]. An unrooted phylogenetic tree was constructed from the whole genome sequence alignment using the Maximum Parsimony method and MEGA5 software [25]. To test, whether the mosaic character of identified loci were a result of intra-strain recombination, potential donor sites were screened from the entire Bosnia A genome using several computer programs and algorithms including RDP3 [26], EditSeq software (DNASTAR, Madison, WI, USA), BLAST (http://blast.ncbi.nlm.nih.gov), and Crossmatch (http://www.phrap.org/phredphrapconsed.html). We failed to find any potential donor sites in the Bosnia A genome. We also failed to find any TPA- or TPE-specific NGS reads in the regions having a mosaic character.

### Nucleotide sequence accession number

The complete genome sequence of the Bosnia A strain was deposited in the GenBank under accession number CP007548.

## Results

### Whole genome sequencing, genome parameters, gene annotation

Sequencing of the TEN Bosnia A strain genome using three independent next-generation sequencing platforms yielded a total combined average coverage of 513×. The summarized genomic features of the Bosnia A strain in comparison to previously sequenced TPA and TPE strain genomes are shown in Table 1. The size of the Bosnia A genome (1,137,653 bp) was 1,628–2,828 bp shorter than the sizes of previously published genomes for TPA and TPE strains [10]–[15]. The overall gene order in the Bosnia A genome was identical to other TPE and TPA strains. Altogether, 1125 genes were annotated in the Bosnia A genome including 54 untranslated genes encoding rRNAs, tRNAs and other ncRNAs (short bacterial RNA molecules that are not translated into proteins). A total of 640 genes (56.9%) encoded proteins with predicted function, 137 genes encoded treponemal conserved hypothetical proteins (TCHP, 12.2%), 141 genes encoded conserved hypothetical proteins (CHP, 12.5%), 145 genes encoded hypothetical proteins (HP, 12.9%) and 8 genes (TENDBA_0082a, TENDBA_0146, TENDBA_0316, TENDBA_0370, TENDBA_0520, TENDBA_0532, TENDBA_0812 and TENDBA_1029; 0.7%) were annotated as pseudogenes. The average and median gene lengths of the Bosnia A genome were calculated to 979.2 bp and 831 bp, respectively. The intergenic regions covered 52.6 kbp and represented 4.63% of the total Bosnia A genome length. In general, other calculated genomic parameters were similar to other TPE strains.

**Table 1:** Summary of the genomic features of the *Treponema pallidum* subsp. *endemicum* Bosnia A strain and four *T. pallidum* subsp. *pertenue* strains (Samoa D, CDC-2, Gauthier and Fribourg-Blanc).

Genome parameter	Bosnia A	Fribourg-Blanc^a^	Samoa D^b^	CDC-2^b^	Gauthier^b^
GenBank accession number	CP007548.1	CP003902.1	CP002374.1	CP002375.1	CP002376.1
Genome size (bp)	1,137,653	1,140,481	1,139,330	1,139,744	1,139,417
G+C content (%)	52.77	52.80	52.80	52.80	52.80
Intergenic region length (bp) (% of the genome length)	52,643 (4.63)	52,785 (4.63)	52,844 (4.64)	52,963 (4.65)	53,300 (4.68)
Average/median gene length (bp)	979.2/831.0	982.6/831.0	980.3/831.0	980.4/831.0	979.3/831.0
No. of predicted protein-encoding genes	1063	1065	106810681068		
No. of genes encoded on plus/minus DNA strand	600/525	599/523	600/525600/525600/525		
No. of genes coding for proteins with predicted function	640	640	640640640		
No. of genes coding for treponemal conserved hypothetical proteins	137	139	140140140		
No. of genes coding for conserved hypothetical proteins	141	141	141141141		
No. of genes coding for hypothetical proteins	145	145	147147147		
No. of annotated pseudogenes (no. of pseudogenes when compared to the Nichols CP004010.2 genome sequence^c^)	8 (16)	3 (14)	3 (12)3 (12)3 (12)		
No. of tRNA loci	45	45	454545		
No. of rRNA loci	6 (2 operons)	6 (2 operons)	6 (2 operons)6 (2 operons)6 (2 operons)		
No. of ncRNAs	3	3	333		

a [15].

b [11].

c in previous studies [11], [15], Samoa D, CDC-2, Gauthier and Fribourg-Blanc genomes were compared to the Nichols CP004010.2 genome sequence [14].

When compared to TPA strains, the Bosnia A genome contained a 635 bp long insertion in the *tprF* locus. In this respect, the Bosnia A genome was similar to TPE strains. When compared to both TPA and TPE genomes, the Bosnia A genome contained a 2300 bp long deletion involving the *tprF* and *G* loci (TPANIC_0316 and TPANIC_0317 in the Nichols genome CP004010.2 [14]). Moreover, the predicted TENDBA_0316 gene (1860 bp in length) was a chimera encompassing the *tprG* 5′-region, *tprI*-like sequence and the *tprF* 3′-region, and was hence designated as *tprGI* as previously described by Centurion-Lara et al. [27] (Table 2). Two insertions of 65 bp and 52 bp, respectively, resulted in the prediction of two hypothetical genes, TENDBA_ 0126b and TENDBA_548a. The same orthologs were also predicted in TPE but not in TPA strains (Table 2).

**Table 2:** Frameshift mutations and substitutions resulting in significant protein truncations, elongations and novel annotations in the Bosnia A genome in comparison with TPA and TPE strains.

Gene Predicted protein/Function^a^	Nucleotide difference in Bosnia A strain	Difference in comparison with	Identity to	Coordinates of the difference in the Bosnia A genome (CP007548.1)	Result of the frameshift mutation/substitution
TENDBA_0009	2 bp insertion^b^	TPA strains	TPE strains	9428–9429	reverted frameshift mutation
TprA/Virulence					functional *tprA* gene
TENDBA_0082a	1 bp insertion	TPA/TPE strains	Bosnia A-specific	92250	frameshift mutation, significant protein truncation
HP/Unknown					gene annotated as pseudogene
TENDBA _0103	1 bp deletion	TPA strains	TPE strains	113544–113545	reverted frameshift mutation
RecQ/DNA replication, repair and recombination					functional *recQ* gene
TENDBA_0126b	65 bp insertion	TPA strains	TPE strains	149005–149069	frameshift mutation, gene elongation
HP/Unknown	1 bp deletion	TPA/TPE strains	Bosnia A-specific	149363–149364	prediction of TENDBA_0126b gene^c^
TENDBA _0314	1 bp deletion	TPA strains	TPE strains	331583–331584	frameshift mutation
TCHP/Unknown					fusion of genes orthologous to TPA genes TP0314 and TP0315 (813 bp)
TENDBA _0316	635 bp insertion	TPA strains	TPE strains	332334–332968	reverted frameshift mutation, chimeric tprGI^d^
TprGI/Virulence	2300 bp deletion	TPA/TPE strains	Bosnia A-specific	333273–333274	gene annotated as pseudogene
TENDBA_0370	1 bp substitution	TPA strains	TPE strains	394198	internal stop codon generation, significant protein truncation
HP/Unknown					gene annotated as pseudogene
TENDBA _0548a	52 bp insertion	TPA strains	TPE strains	592543–592594	frameshift mutation
HP/Unknown					prediction of TENDBA_0548a gene^c^
TENDBA _0671	1 bp substitution	TPE strains	TPA strains	736075	alternative start codon generation, gene elongation
Ethanolamine-phosphotransferase/General metabolism					
TENDBA _0911a	1 bp deletion	TPA strains	TPE strains	989806–989807	frameshift mutation
HP/Unknown					prediction of TENDBA_0911a gene^c^
TENDBA _1029	2 bp deletion^b^	TPA/TPE strains	Bosnia A-specific	1123143–1123144	frameshift mutation, significant protein truncation
TCHP/Unknown					gene annotated as pseudogene
TENDBA _1031	378 bp insertion	TPE strains	TPA strains	1123623–1124000	frameshift mutation, gene elongation^d^
TprL/Virulence	(including 1 bp deletion specific for Bosnia A^b^)			1123700–1123701	

a HP – hypothetical protein, TCHP – treponemal conserved hypothetical protein.

b nucleotide differences in the regions containing simple sequence repeats (short tandem repeats); when compared, *tprA* gene was functional in Bosnia A and TPE strains and not among TPA strains (except for strain Sea 81-4; see [37]).

c due to same nucleotide differences (indels), orthologous genes to TENDBA_0126b, TENDBA_0548a and TENDBA_0911a genes were previously predicted in all TPE strains when compared to the TPA genomes.

d [27].

Besides the annotated pseudogenes in the Bosnia A genome (see above), 8 additional genes (orthologous to TP0129, TP0132, TP0135, TP0266, TP0318, TP0370, TP0671 and TP1030) were considered pseudogenes. The same genes were also considered pseudogenes in TPE strains [11], [15] (Table 1).

### Similarity of the Bosnia A genome to the available TPA and TPE genomes

**Figure 1:**
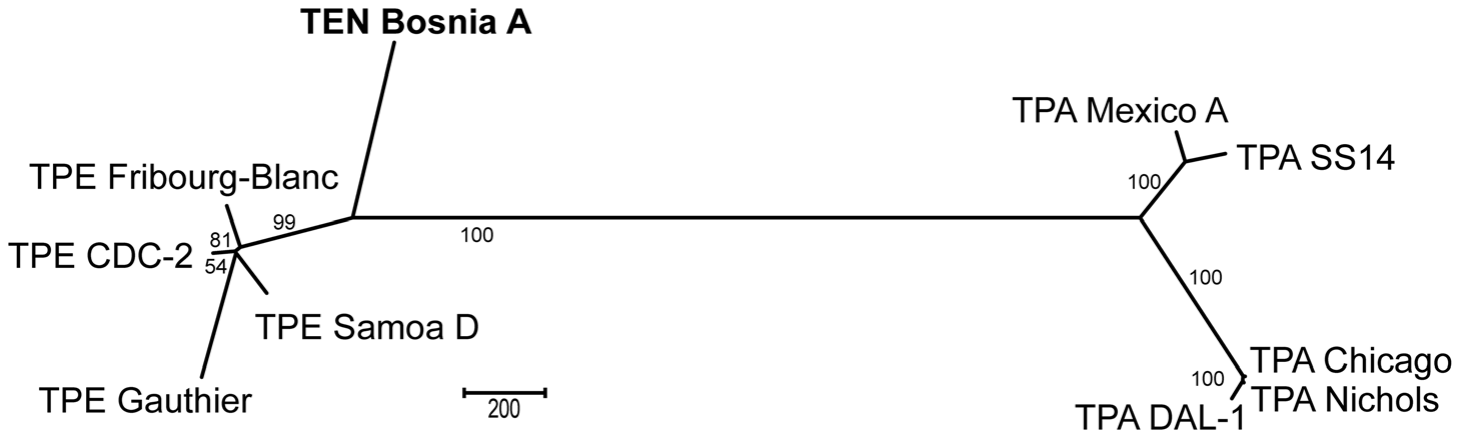
Unrooted tree based on the alignment of the Bosnia A genome with additional treponemal genomes. An unrooted tree was constructed from the complete genome sequences of TPA strains (Nichols, Chicago, DAL-1, SS14, and Mexico A), TPE strains (CDC-2, Gauthier, Samoa D, and Fribourg-Blanc), and the TEN strain (Bosnia A) using the Maximum Parsimony method and MEGA5 software [25]. The bar scale corresponds to a difference of 200 nucleotides. Bootstrap values based on 1,000 replications are shown next to the branches. All positions containing indels in at least one genome sequence were omitted from the analysis. There were a total of 1,128,391 nucleotide positions aligned in the final dataset.

Sequence relatedness of the Bosnia A genome to other *Treponema pallidum* genomes is shown in Fig. 1. This unrooted tree was constructed using several available whole genome sequences of uncultivable pathogenic treponemes. The image clearly showed clustering of the Bosnia A strain with the TPE strains. The Bosnia A genome was found to be 99.91–99.94% and 99.79–99.82% identical to the TPE and TPA genomes, respectively (Table 3). The nucleotide diversity between TPE strains and the Bosnia A strain (0.00063±0.00032 to 0.00086±0.00043) was about three times lower than the nucleotide diversity between TPA strains and the Bosnia A strain (0.00181±0.00090 to 0.00212±0.00106). For comparison, calculated π values between the Bosnia A strain and individual TPA strains were of the same order of magnitude as π values between TPA and TPE strains (Table 4).

**Table 3:** Calculated nucleotide identity and nucleotide diversity (π ± standard deviation) between Bosnia A strain and individual TPA and TPE strains^a^.

Strain	Nucleotide identity (%)	Nucleotide diversity (π ± SD)
TPA Nichols	99.792	0.00209±0.00104
TPA DAL-1	99.788	0.00212±0.00106
TPA Chicago	99.793	0.00207±0.00103
TPA SS14	99.813	0.00187±0.00094
TPA Mexico A	99.819	0.00181±0.00090
TPE Samoa D	99.932	0.00068±0.00034
TPE CDC-2	99.937	0.00063±0.00032
TPE Gauthier	99.914	0.00086±0.00043
TPE Fribourg-Blanc	99.931	0.00069±0.00034

a All positions containing indels in at least one genome sequence were omitted from the analysis. There were a total of 1,128,391 nucleotide positions aligned in the final dataset.

**Table 4:** Calculated nucleotide diversity (π ± standard deviation) between TPA and TPE strains, within individual TPE strains, within TPA strains, and between Bosnia A strain and TPA and TPE strains.

Strains	Nucleotide diversity (π ± SD)
TPA strains vs. TPE strains	0.00166±0.00083 to 0.00209±0.00104
TPE strains	0.00016±0.00008 to 0.00044±0.00022
TPA strains	0.00003±0.00002 to 0.00070±0.00035
Bosnia A strain vs. TPA strains	0.00181±0.00090 to 0.00212±0.00106
Bosnia A strain vs. TPE strains	0.00063±0.00032 to 0.00086±0.00043

### Bosnia A specific sequences

**Figure 2:**
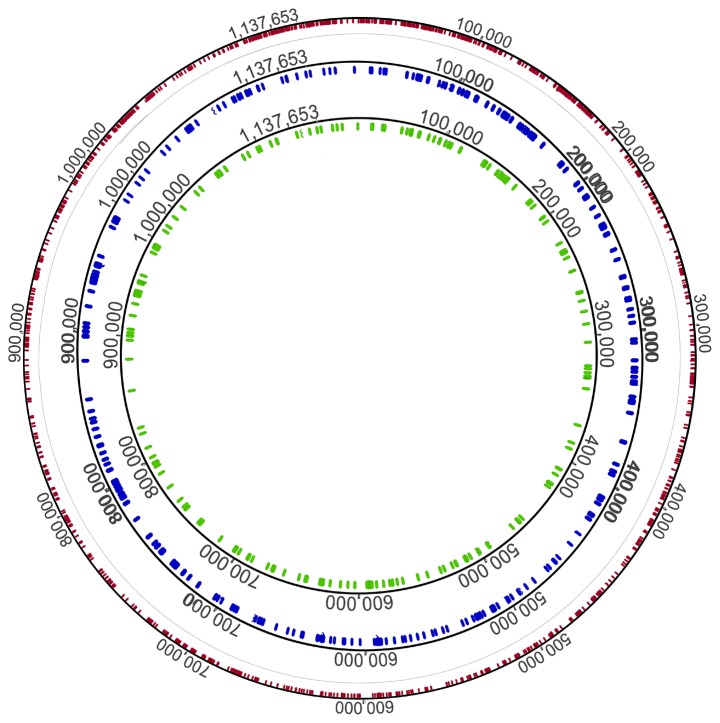
Representation of the Bosnia A chromosome with location of Bosnia A-, TPE-, and TPA-specific sequences. Bosnia A-specific sequences are shown in green while TPE-specific sequences (TPA and Bosnia A sequences are identical in these loci) are shown in blue. TPA-specific sequences (TPE and Bosnia A sequences are identical in these loci) are shown in red. Bosnia A-specific sequences comprised 406 loci (encompassing a total of 2772 bp) while TPE- and TPA-specific sequences were found in 197 (635 bp) and 1422 (2335 bp) loci, respectively.

To identify Bosnia A-specific differences, the Bosnia A genome was compared to the available genomes of TPE strains [11], [15] and TPA strains [10], [12]–[14]. The Bosnia A strain-specific sequences were defined as those not present in both TPA and TPE strains and altogether comprised 406 differences (indels and substitutions with a total length of 2772 bp) equally distributed along the Bosnia A genome (Fig. 2). Differences in coding regions included 9 deletions, 5 insertions and 360 nucleotide substitutions for a total of 2728 bp (Table 5). Those 360 substitutions resulted in 197 Bosnia A-specific amino acid differences in the putative proteome. Most of the nucleotide substitutions were found in the TENDBA_0136, TENDBA_0548, TENDBA_0856, TENDBA_0859 and TENDBA_0865 genes (Table 5). Bosnia A-specific frameshift mutations (caused by three deletions and one insertion) resulted in significant gene truncation (TENDBA_0082a, TENDBA_0316 and TENDBA_1029) or elongation (TENDBA_0126b) (Table 2). Other detected indels resulted in 6 protein shortenings (TENDBA_0067, TENDBA_0136, TENDBA_0225, TENDBA_0548, TENDBA_0859, and TENDBA_0865) and 4 protein elongations (TENDBA_0856, TENDBA_0859, TENDBA_0897, and TENDBA_0898) (Table 5).

**Table 5:** Genome differences specific for the TEN Bosnia A strain^a^.

	Non-coding sequences (altogether 44 bp)		Coding sequences (altogether 2728 bp)				
Nucleotide difference	Number of differences	Number of affected nucleotides	Number of differences	Number of affected nucleotides	Affected gene	Predicted protein^b^	Function
deletion	1	1×13 bp	9	1× single bp	TENDBA_0126b^c^	HP	Unknown
		(altogether 13 bp)		1×2 bp	TENDBA_1029^c^	TCHP	Unknown
				1×2300 bp	TENDBA_0316^c^	TprGI	Virulence
				(altogether 2303 bp of protein truncation or elongation)			
				2×3 bp	TENDBA_0136^d^	TCHP	Virulence
					TENDBA_0225	CHP	Unknown
				1×4 bp	TENDBA_0548	TCHP	Unknown
				1×6 bp	TENDBA_0859	TCHP	Unknown
				1×9 bp	TENDBA_0067	CHP	Unknown
				1×24 bp	TENDBA_0865	TCHP	Unknown
				(altogether 49 bp of protein shortening)			
insertion	0		5	1× single bp	TENDBA_0082a^c^	HP	Unknown
				(altogether 1 bp of protein truncation due to frameshift mutation)			
				3×3 bp	TENDBA_0859 (2x)	TCHP	Unknown
					TENDBA_0898	RecB	DNA replication, repair and recombination
				1×6 bp	TENDBA_0856	TCHP	Unknown
				(altogether 15 bp of protein elongation)			
substitution	31		356	197 different aa	TENDBA_0136^d^	TCHP	Virulence
			(TPA identical with TPE)	122 identical aa	TENDBA_0548	TCHP	Unknown
			4		TENDBA_0856	TCHP	Unknown
			(TPA different from TPE)		TENDBA_0859	TCHP	Unknown
					TENDBA_0865	TCHP	Unknown

a The *tprK* gene (TENDBA_0897) was excluded from this list of differences because of high intra-strain sequence diversity.

b HP – hypothetical protein, CHP – conserved hypothetical protein, TCHP – treponemal conserved hypothetical protein.

c see also Table 2.

d TPA and TPE orthologs to TENDBA_0136 have been experimentally shown to bind human fibronectin [28].

All affected genes code for hypothetical proteins of unknown function except for TENDBA_0898 coding for RecB (exodeoxyribonuclease V beta subunit; EC3.1.11.5). TENDBA_0136 and TENDBA_0865 have been predicted to be putative outer membrane proteins. In addition, TPA and TPE orthologs to TENDBA_0136 have been experimentally shown to bind human fibronectin [28]. TENDBA_0856 has been predicted to be putative lipoprotein. No putative conserved domains have been detected in hypothetical proteins except for TENDBA_0067, TENDBA_0225 and TENDBA_1029 containing TPR (tetratricopeptide) domain, LRR_5 (leucine rich repeat) domain and DbpA (RNA binding) domain, respectively (Table 5). All nonsynonymous substitutions have been identified outside the predicted domains.

### Bosnia A sequences shared with TPE but not TPA strains

Genome sequences differentiating the Bosnia A strain from the TPA but not TPE strains are shown in Fig. 2. These sequences were found to be regularly distributed along the Bosnia A genome and altogether comprised 1422 differences (indels and substitutions of total length of 2335 bp). In the coding regions, 2128 bp including 13 deletions, 9 insertions and 1296 substitutions differentiated genomes of TPA strains from Bosnia A and other TPE strains (Table 6). A set of 1296 substitutions resulted in 631 amino acid differences in the encoded proteins. Most of the differences were found in genes TENDBA_0117 (*tprC*), TENDBA_0131 (*tprD*), TENDBA_0133, TENDBA_0134, TENDBA_0136, TENDBA_0304, TENDBA_0314, TENDBA_0462, TENDBA_0619, TENDBA_0620 (*tprI*), and TENDBA_0621 (*tprJ*) (Table 6).

**Table 6:** Genome sequences of Bosnia A strain identical to TPE strains and different from TPA strains^a^.

	Non-coding sequences (altogether 207 bp)		Coding sequences (altogether 2128 bp)				
Nucleotide difference	Number of differences	Number of affected nucleotides	Number of differences	Number of affected nucleotides	Affected gene	Predicted protein^b^	Function
deletion	8	1× single bp	13	3× single bp	TENDBA_0103^c^	RecQ	DNA replication, repair and recombination
		1×2 bp		(altogether 3 bp of protein elongation)	TENDBA_0314^c^	TCHP	Unknown
		1×6 bp			TENDBA_0911a^c^	HP	Unknown
		1×9 bp		1×9 bp	TENDBA_0067	CHP	Unknown
		1×13 bp		1×27 bp	TENDBA_0461a	HP	Unknown
		1×17 bp		8×3 bp	TENDBA_0027	HlyC	Cell processes
		1×30 bp		(altogether 60 bp of protein shortening)	TENDBA_0136^d^	TCHP	Virulence
		1×33 bp			TENDBA_0304	TCHP	Unknown
		(altogether 111 bp)			TENDBA_0314	TCHP	Unknown
					TENDBA_0619	TCHP	Unknown
					TENDBA_0621 (2x)	TprJ	Virulence
					TENDBA_0856a/TENDBA_0858	HP/TCHP	Unknown/Unknown
insertion	3	3× single bp	9	1×2 bp	TENDBA_0009^c^	TprA	Virulence
		(altogether 3 bp)		1×52 bp	TENDBA_0548a^c^	HP	Unknown
				1×65 bp	TENDBA_0126b^c^	HP	Unknown
				1×635 bp	TENDBA_0316^c^	TprGI	Virulence
				(altogether 754 bp of protein truncation or elongation due to frameshift mutations)			
				5×3 bp	TENDBA_0129a/TENDBA_0129b	HP/HP	Unknown/Unknown
				(altogether 15 bp of protein elongation)	TENDBA_0462 (2x)	CHP	Unknown
					TENDBA_0856a/TENDBA_0858	HP/TCHP	Unknown/Unknown
					TENDBA_0967	TCHP	Unknown
substitution	93		1296	631 different aa	TENDBA_0117	TprC	Virulence
				427 identical aa	TENDBA_0131	TprD	Virulence
					TENDBA_0133	TCHP	Unknown
					TENDBA_0134	TCHP	Unknown
					TENDBA_0304	TCHP	Unknown
					TENDBA_0314	TCHP	Unknown
					TENDBA_0462	CHP	Unknown
					TENDBA_0619	TCHP	Unknown
					TENDBA_0620	TprI	Virulence
					TENDBA_0621	TprJ	Virulence

a The *tprK* gene (TENDBA_0897) was excluded from this list of differences because of high intra-strain sequence diversity.

b HP – hypothetical protein, CHP – conserved hypothetical protein, TCHP – treponemal conserved hypothetical protein.

c see also Table 2.

d TPA and TPE orthologs to TENDBA_0136 have been experimentally shown to bind human fibronectin [28].

Except for TENDBA_0103 coding for RecQ (ATP-dependent DNA helicase; EC3.6.4.12) and TENDBA_0027 coding for HlyC (putative hemolysin), all other affected genes code for hypothetical proteins of unknown function. TENDBA_0134 has been predicted to be putative outer membrane protein. TENDBA_0462 and TENDBA_0858 have been predicted to be putative lipoproteins. No putative conserved domains have been detected in hypothetical proteins except for TENDBA_0067 and TENDBA_0304 conatining TPR (tetratricopeptide) domain and peptidase_MA_2 domain, respectively (Table 6). All nonsynonymous substitutions have been identified outside the predicted domains.

### Bosnia A sequences shared with TPA but not TPE strains

Genome sequences differentiating the Bosnia A strain from TPE but not TPA strains are shown in Fig. 2. These sequences were also found to be regularly distributed along the Bosnia A genome and, altogether, comprised 197 differences in genome positions (containing indels and substitutions encompassing a total of 635 bp). Three deletions, three insertions and 174 substitutions (Table 7) were found within the Bosnia A coding regions, encompassing a total of 612 bp. The 174 substitutions resulted in 101 amino acid differences in the putative encoded proteins. Most of the substitution differences were found in genes TENDBA_0136, TENDBA_0488, TENDBA_0577, TENDBA_0856a/TENDBA_0858, TENDBA_0859, TENDBA_0865 and TENDBA_0968 (Table 7). An insertion of 378 bp in TENDBA_1031 (*tprL*) resulted in a gene elongation (Table 2).

**Table 7:** Genome sequences of Bosnia A strain identical to TPA strains and different from TPE strains^a^.

	Non-coding sequences (altogether 23 bp)		Coding sequences (altogether 612 bp)				
Nucleotide difference	Number of differences	Number of affected nucleotides	Number of differences	Number of affected nucleotides	Affected gene	Predicted protein^b^	Function
deletion	1	1×6 bp	3	1×3 bp	TENDBA_0856a/TENDBA_0858	HP/TCHP	Unknown/Unknown
		(altogether 6 bp)		1×9 bp	TENDBA_0859	TCHP	Unknown
				1×12 bp	TENDBA_0577	TCHP	Unknown
				(altogether 24 bp of protein shortening)			
insertion	1	1×2 bp	3	1×6 bp	TENDBA_0548	TCHP	Unknown
		(altogether 2 bp)		1×30 bp	TENDBA_0136^c^	TCHP	Virulence
				1×378 bp	TENDBA_1031^d^	TprL	Virulence
				(altogether 414 bp of protein elongation)			
substitution	15		174	101 different aa	TENDBA_0136^c^	TCHP	Virulence
				48 identical aa	TENDBA_0488	Mcp	Cell processes
					TENDBA_0577	TCHP	Unknown
					TENDBA_0856a/TENDBA_0858	HP/TCHP	Unknown/Unknown
					TENDBA_0859	TCHP	Unknown
					TENDBA_0865	TCHP	Unknown
					TENDBA_0968	TCHP	Unknown

a The *tprK* gene (TENDBA_0897) was excluded from this list of differences because of high intra-strain sequence diversity.

b HP – hypothetical protein, TCHP – treponemal conserved hypothetical protein.

c TPA and TPE orthologs to TENDBA_0136 have been experimentally shown to bind human fibronectin [28].

d see also Table 2.

TENDBA_0488 codes for Mcp (methyl-accepting chemotaxis) protein. All other genes code for hypothetical proteins of unknown function. Two genes have been predicted to encode putative outer membrane proteins (TENDBA_0136 and TENDBA_0865) and one gene has been predicted to encode putative lipoprotein (TENDBA_0858). No putative conserved domains have been detected in hypothetical proteins (Table 7).

### Several genetic loci of the Bosnia A genome show striking similarity to TPA sequences

**Figure 3:**
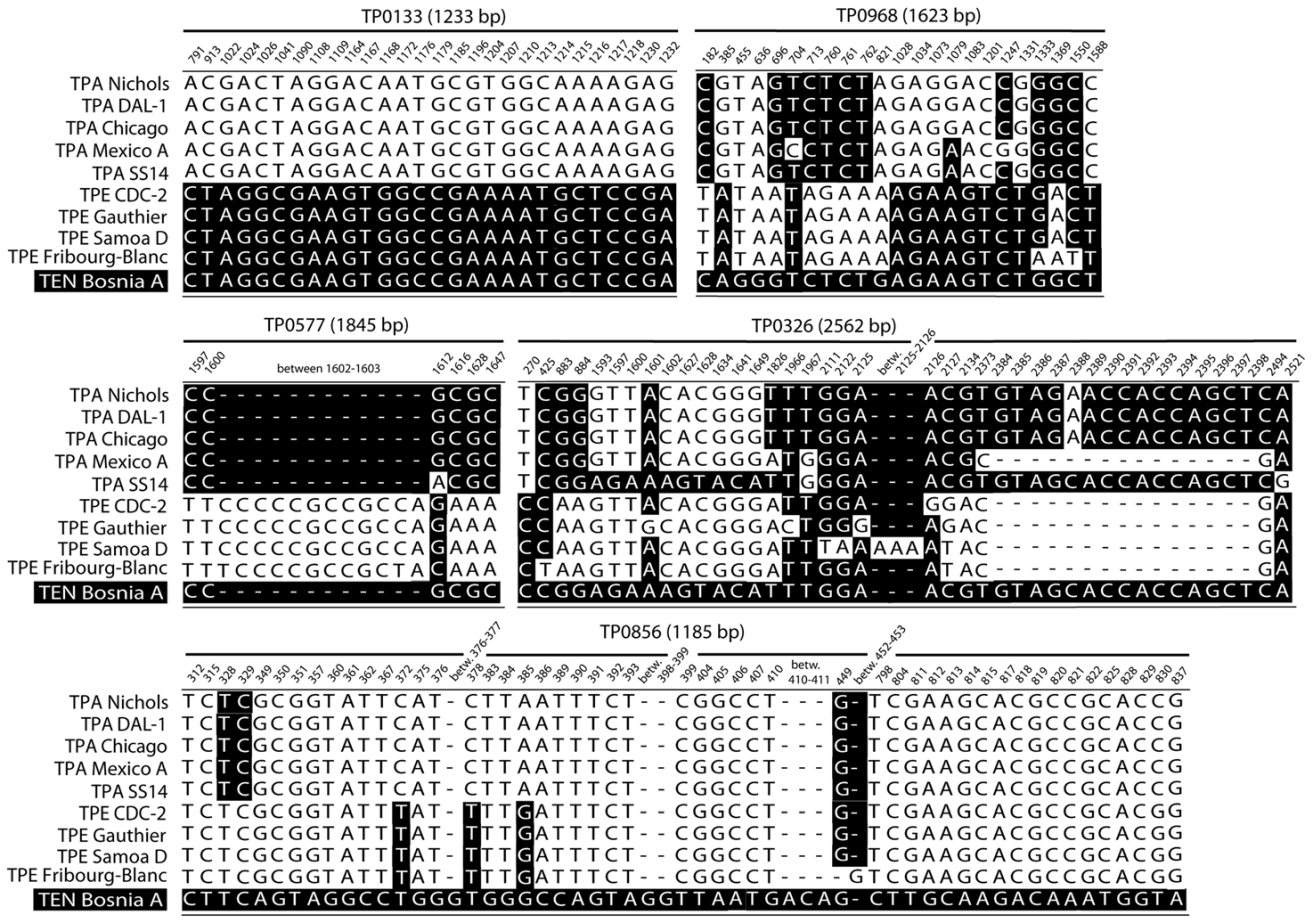
Sequence alignments of TENDBA_0133, TENDBA_0968, TENDBA_0577, TENDBA_0326 and TENDBA_0856 loci with the orthologous sequences of TPA and TPE genomes. Sequences of five TPA strains (Nichols, DAL-1, Chicago, Mexico A and SS14) and four TPE strains (CDC-2, Gauthier, Samoa D and Fribourg-Blanc) are shown. Numbers above the alignment represent gene coordinates in the re-sequenced TPA Nichols strain (CP004010.2 [14]). While the alignment of TENDBA_0133 showed locus completely identical to TPE strains, TENDBA_0968, TENDBA_0577 and TENDBA_0326 loci showed the presence of TPA sequences in the genome of Bosnia A. The TENDBA_0856 locus represent Bosnia A specific region with sporadic nucleotide positions identical to TPA sequences. The TPE-like sequence was found in most of the Bosnia A loci while the pattern found in TENDBA_0968 was also found in TENDBA_0858 (not shown) and the pattern identified in the TENDBA_0577 was found also in the TENDBA_1031 (not shown). The alignment pattern in TENDBA_0326 was previously found in TENDBA_0488 [12] and the pattern in TENDBA_0856 in TENDBA_0865.

Despite the overall sequence similarity of the Bosnia A genome to TPE strains, several chromosomal sequences were found to be almost identical to sequences in TPA strains. The Bosnia A sequence in the TENDBA_0577 locus was identical to four out of 5 orthologous sequences of completely sequenced TPA strains (Fig. 3). In the TENDBA_0968 locus, stretches of TPA- and TPE-like sequences were found (Fig. 3) and a similar pattern was also found in TENDBA_0858 (not shown). In addition, TENDBA_0326 (*tp92*, *bamA*) was identical to the orthologous sequence of TPA SS14 (coordinates 1593–1649, Fig. 3) and to all TPA strains (with the exception of the TPA Mexico A strain) between coordinates 2127–2494. The TPA Mexico A strain is, in this region, similar to TPE strains [12], [29]. While the latter TPA-like sequences in TENDBA_0326 were almost 0.4 kbp long, other TPA-like sequences were usually relatively short, ranging from about 50–70 bp. However, TPA-like sequences of the Bosnia A strain were clearly different from Bosnia A-specific sequences with sporadic nucleotide positions identical to TPA sequences (TENDBA_0856; Fig. 3). The previously reported 378 bp insertion almost identical to TPA strains (differing only in one nucleotide position [27]) was confirmed in TENDBA_1031 as well as the nucleotide mosaic in the TP0488 (*mcp2-1*) locus; revealing a sequence identical to TPA Mexico A (with the exception of 2 single nucleotide substitutions [12]). Altogether, at least seven TPA-like sequences having 5 or more nucleotide positions identical to TPA sequences and not interrupted by TPE-like nucleotide positions were found in the Bosnia A genome.

## Discussion

The first complete genome sequence of the bejel-causing agent, *T. pallidum* subsp. *endemicum* (TEN) strain Bosnia A, was determined using three independent next-generation sequencing techniques. Because the total combined coverage was >500× and all sequencing ambiguities were resolved with Sanger sequencing, the quality of this new genome is very high. This allowed us to carry out a comparative analysis of the Bosnia A genome with the already available treponemal genomes [10]–[15], [30] with a high degree of confidence that our results would not be affected by sequencing errors. In several of the previously published genomes, the whole genome sequence was compared to whole genome fingerprinting data to assess the quality of the genome sequence. In each of the previously tested genomes, the sequencing error rate was less than 10^−4^ [11], [12], [15], [30].

The genome length of strain Bosnia A (1,137,653 bp) is about 2 kbp shorter than the length of TPE or TPA genomes. This is caused by a 2300 bp deletion in the *tprF* and *tprG* loci. This deletion was also confirmed in the TEN Iraq B sequence [27] suggesting that this is a common feature of bejel strains. An identical deletion was also found in the *T. paraluisleporidarum* ec. Cuniculus genome (formerly denoted *T. paraluiscuniculi* Cuniculi A [30], [31]). Moreover, this type of deletion was observed during PCR amplification of the *tprF* and *tprG* loci in other treponemal genomes (M. Strouhal, D. Šmajs; unpublished data). This fact, together with the presence of repeats in the flanking regions suggests that this 2300 bp deletion is a result of polymerase slippage and that this deletion could have happened several times independently during evolution. In fact, no other similarities between the Bosnia A and *T. p.* ec. Cuniculus genome were found with respect to other identified indels in the *T. p.* ec. Cuniculus genome.

The overall genetic similarity of Bosnia A to the sequenced TPE strains is 99.91–99.94%, at the DNA level. For comparison, the sequence similarity between TPA and TPE strains is greater than 99.8% [11], [15]. This enormous sequence similarity among TPA, TPE and TEN strains is the molecular basis for the long established fact that individual etiological agents of syphilis and endemic treponematoses (yaws and bejel) cannot be distinguished by their morphology or serology.

Although syphilis, yaws, and bejel show differences in their geographical distribution, mode of transmission, invasiveness and pathogenicity, it is known that the clinical symptoms of these diseases overlap and one disease can mimic the others. Interestingly, in very dry areas, yaws symptoms are almost the same as bejel symptoms [32]; which again reflects the extremely high sequence similarity between TPE and TEN strains. In many or perhaps most cases, the final diagnosis is therefore often based on the epidemiological context of the infection. However, at the same time, even small genomic differences (although not known at present) have the potential to influence the phenotypic differences between the clinical manifestations of syphilis, yaws and bejel. Additional whole genome sequences of TPA, TPE and TEN strains will help to identify a set of invariant differences between the etiological agents of these diseases, which could help answer this question.

At the same time, the TEN Bosnia A strain is clearly distant from the cluster of TPE strains. However, additional TEN whole genome sequences will be needed to assess the variability within TEN strains. To our knowledge, there is only one additional laboratory stock of TEN, i.e. strain Iraq B. Previous studies on the Iraq B isolate revealed a high degree of similarity to Bosnia A [27], [29], [33]–[36] suggesting that this strain is more related to Bosnia A than to TPE strains.

Most prominent genetic changes between Bosnia A and TPE and/or TPA genomes resulting in protein truncations or elongations were located in just 14 genes. These genes encoded TprA, F, G, and L proteins, RecQ protein, ethanolamine phosphotransferase, and treponemal conserved hypothetical proteins (3) or hypothetical proteins (5). Both Tpr and RecQ proteins were found to also be affected in the *T. p.* ec. Cuniculus genome [30]. While the *tprA* gene was functional in Bosnia A and TPE strains but not among TPA strains (except for strain Sea 81-4; see [37]), *tprF* and *tprG* were partially deleted (similarly to *T. p.* ec. Cuniculus genome) and the *tprL* gene was elongated in a way that was similar to that seen in TPA strains. These changes were already described in detail by Centurion-Lara et al. [27]. Tpr proteins likely play an important role in treponemal infectivity, pathogenicity, immune evasion and host specificity. Tpr proteins induce an antibody response during infection and exhibit heterogeneity both within and among *T. pallidum* subspecies and strains [38]–[40]. In the *T. p.* ec. Cuniculus genome, a mutation in *recQ* resulted in a predicted RecQ protein without a C-terminal or DNA-binding domain [41]; on the other hand in Bosnia A the frameshift reversion led to a functional *recQ* gene (similar to that seen in TPE genomes [11]). Other prominent changes seen in the Bosnia A strain include a different number of tandem repeat units in TENDBA_0433 (encoding Arp) and TENDBA_0470 genes (encoding conserved hypothetical protein) compared to orthologous genes in individual TPE and TPA strains. The same number of 60-bp tandem repeat units (all of Type II) within the *arp* gene was found in the Bosnia A genome as previously described [42]. Variable numbers of tandem repeat units in genes orthologous to TENDBA_0470 have already been described in TPE and TPA strains [11], [15], [19].

The genome of Bosnia A showed several genetic loci with sequences identical to TPA sequences (Fig. 3). The TENDBA_0577 gene encoded treponemal conserved hypothetical protein of unknown function with predicted cytoplasmic membrane localization. This gene was completely identical to TPA orthologs and differed from TPE orthologs by deletion of 12 nucleotides and substitution of 5 nucleotides. Recent studies of σ factor RpoE (TP0092) binding sites identified gene TP0577 (orthologous to TENDBA_0577) as one of 22 putative TP0092-controlled ORFs [43]. The TENDBA_0577 thus could possibly code for a protein integrated in the stress response pathway during the first days post infection. Similarly, the 378 bp insertion in TENDBA_1031 is with exception of a 1 nucleotide insertion almost identical to orthologs of the TPA strain (but not to TPE strains). In other genes (TENDBA_0968, TENDBA_0858), 50–70 bp long sequences identical to one or several TPA strains were found indicating that the genome of Bosnia A incorporated sequences identical to TPA strains. Most of the above mentioned genes were found to evolve under positive selection in TPA-TPE comparisons [11]. In fact, previous papers found this type of mixed TPA and TPE sequences in TPA Mexico A and South Africa strains [12], [29]. Moreover, previous reports have shown that TEN strain Bosnia A contains the same nucleotide mosaic at the TP0488 (*mcp2-1*) locus as TPA Mexico A (with the exception of 2 single nucleotide substitutions). Despite the numerous efforts to identify potential donor sites within TPA Mexico A that could explain the existence of these sequences by intra-strain recombination [12], no such sites have been identified in the Mexico A genome. Similarly, no donor sites have been identified in the Bosnia A genome either. It is likely that these sequences identical to TPA in the Bosnia A genome could result from inter-strain recombination event between TPA and TEN strains during a simultaneous infection of multiple hosts during the TEN evolution. Although the overall genome sequence of Bosnia A is related to TPE strains, horizontal gene transfer appears to be the mechanism that introduced at least seven chromosomal sequences related to TPA SS14, TPA Mexico A, and other TPA strains. In fact, both the TPA SS14 and Mexico A sequences are required and sufficient to provide sequences to Bosnia A genome. Moreover, at least two subsequent transfers had to occur to introduce both SS14- and Mexico A-specific sequences. Experimental infection with either TPA, TPE or TEN strains did not result in complete cross-protection [9]. In addition, recombination mechanisms are more active during treponemal infection and represent important genetic mechanisms for avoiding the host immune response [40]. Moreover, the absence of modification and restriction systems and the presence of genes for homologous recombination in pathogenic treponemes [16] appear to allow incorporation of foreign DNA molecules with subsequent integration into chromosomal DNA. Therefore, uptake of TPA DNA by a TEN strain during a simultaneous infection of multiple hosts appears to be a possible explanation.

It is clear that TPA strains can be classified as SS14-like (SS14, Mexico A) and Nichols-like strains (Nichols, DAL-1, Chicago) [14], [44] and that most of the TPA strains causing infections throughout the world are in fact SS14-like strains [36]. However, it is not clear if the SS14 and Mexico A sequences in the Bosnia A genome reflect a greater prevalence of SS14-like strains in the human population or an accidental coincidence of transfers from SS14-like strains. Moreover, there are several loci in the Bosnia A genome similar to the TENDBA_0856 locus (TENDBA_0483, TENDBA_0858, TENDBA_0865) that represent regions of Bosnia A-specific sequences with only sporadic nucleotide positions that are identical to TPA sequences. These sequences may be identical to other, yet unidentified, TPA strains or isolates. If such TPA isolates are identified in the future, they may help to unravel the evolution of TPA and TEN treponemes.

## Supporting Information

Table S1 **Sample preparation of Bosnia A strain for whole genome sequencing using pooled segment genome sequencing (PSGS) strategy.** Sheet 1 (TableS1_BosniaA-primers) contains a list of primers used for whole genome amplification of the Bosnia A strain using PSGS strategy. Sheet 2 (TableS1_BosniaA-overlap reg) contains a list of primers used for amplification of TPI-overlapping regions shorter than 60 bp.
(XLS)
